# Psychological and behavioral differences between low back pain populations: a comparative analysis of chiropractic, primary and secondary care patients

**DOI:** 10.1186/s12891-015-0753-5

**Published:** 2015-10-19

**Authors:** Andreas Eklund, Gunnar Bergström, Lennart Bodin, Iben Axén

**Affiliations:** 1 Karolinska Institutet, Institute of Environmental Medicine, Unit of Intervention and Implementation Research, Nobels väg 13, S-171 77, Stockholm, Sweden; 2 Research Department, Spine Center of Southern Denmark, Hospital Lillebælt and Institute of Regional Health Research, Østre Hougvej 55, DK-5500 Middelfart, Denmark

**Keywords:** Low back pain, Psychological profile, Behavioural profile, Multidimensional pain inventory, MPI, Cluster, Secondary care, Primary care, Chiropractic, Comparative analysis

## Abstract

**Background:**

Psychological, behavioral and social factors have long been considered important in the development of persistent pain. Little is known about how chiropractic low back pain (LBP) patients compare to other LBP patients in terms of psychological/behavioral characteristics.

**Methods:**

In this cross-sectional study, the aim was to investigate patients with LBP as regards to psychosocial/behavioral characteristics by describing a chiropractic primary care population and comparing this sample to three other populations using the MPI-S instrument. Thus, four different samples were compared. A: Four hundred eighty subjects from chiropractic primary care clinics. B: One hundred twenty-eight subjects from a gainfully employed population (sick listed with high risk of developing chronicity). C: Two hundred seventy-three subjects from a secondary care rehabilitation clinic. D: Two hundred thirty-five subjects from secondary care clinics. The Swedish version of the Multidimensional Pain Inventory (MPI-S) was used to collect data. Subjects were classified using a cluster analytic strategy into three pre-defined subgroups (named adaptive copers, dysfunctional and interpersonally distressed).

**Results:**

The data show statistically significant overall differences across samples for the subgroups based on psychological and behavioral characteristics. The cluster classifications placed (in terms of the proportions of the adaptive copers and dysfunctional subgroups) sample A between B and the two secondary care samples C and D.

**Conclusions:**

The chiropractic primary care sample was more affected by pain and worse off with regards to psychological and behavioral characteristics compared to the other primary care sample. Based on our findings from the MPI-S instrument the 4 samples may be considered statistically and clinically different.

**Trial registration:**

Sample A comes from an ongoing trial registered at clinical trials.gov; NCT01539863, February 22, 2012.

## Background

The lifetime prevalence of low back pain (LBP) in Sweden has been found to be 70 % [1]. The long term course of LBP is often associated with persistence and recurrence of pain, a recent systematic review [2] found that 65 % of patients that experience LBP still do so 1 year later. The bio-psycho-social model has become the leading theory of the development and management of chronic pain [3–6]. Psychological [7, 8], behavioral [9] and social factors [3] have been found to be important in the transition from sub-acute into chronic pain [10–13]. Further, psychological characteristics have been found to influence future disability, pain and self-reported improvement after treatment in patients with LBP in primary and secondary care [14–19].

A small number of studies have investigated psychological factors among chiropractic patients and have found little or no correlation with treatment outcome [20–26]. One study showed that chiropractic patients in Sweden had less depression and anxiety compared to other primary care populations [26] . Previous research [27] has shown chiropractic patients to have higher self-rated health, fewer depressive symptoms and lower functional limitations compared to non-chiropractic patients. In line with the above, recent research [28] has investigated primary care patients from GP surgeries (Family Practitioner offices) in Australia and found that the individuals who also saw a chiropractor were less disadvantaged and more likely to suffer from LBP. However, this study also showed that these patients suffered more depression and other chronic health problems as compared to other patient groups in the primary care sector. Thus, the evidence is far from conclusive and more research is needed.

The Multidimensional Pain Inventory (MPI) was originally developed to measure psychological and behavioral aspects of chronic pain [29–31]. It was designed to provide a brief, psychometrically-sound and comprehensive assessment of the chronic pain experience.

The overall aim of this study was to investigate patients with LBP as regards psychosocial and behavioral characteristics. The objectives were: 1) to describe a chiropractic primary care sample of patients with LBP using the MPI instrument, and 2) to compare this sample with three other patient samples, consisting of one sample from gainfully employed industry workers (sick listed with a high risk of developing chronic pain and long term sick leave) and two samples of patients from secondary care sector with long term sick leave due to LBP. It was hypothesized that the chiropractic primary care sample would be similar or better off than the sample from gainfully employed industry workers with regards to the scores from the MPI instrument as they were both gainfully employed populations.

## Methods

### Design

A cross-sectional study design.

### Data materials

The data materials for this study came from four samples of individuals experiencing LBP and/or NP. The first was an ongoing randomized controlled trial and the other three were previously conducted trials.

The data materials were chosen because they were thought to represent distinctly different patient populations with regards to the extent of LBP interference with the subjects’ lives. Possibly, these populations would also display differences in behavioral and psychological profiles indicating clinically meaningful differences across samples. In line with previous research [26, 27], it was hypothesized that the chiropractic primary care population would show a favorable psychological (less affective distress, lower pain severity, less interference and higher level of life control) and behavioral (lower frequency of behaviors by significant others as a response to displays of pain and suffering) profile compared to the secondary care populations but similar or better off than the sample from gainfully employed industry workers (primary care).

### Subjects

The first sample was an ongoing randomized controlled trial [32] investigating a population of patients with LBP from chiropractic primary care clinics in Sweden. The trial started in April 2012 and the inclusion period is expected to take 2 years with a follow-up period of 1 year. The purpose of the RCT was to investigate the effectiveness and cost-effectiveness of preventive manual care (chiropractic maintenance care) for recurrent and persistent LBP. The patients sought care when experiencing acute LBP. Most patients consult their chiropractor directly and pay for the treatment themselves; however a minority is referred and/or may have reimbursement from 3d party payers. Having agreed to participate, subjects were asked to fill in a screening questionnaire. The RCT has been described in detail in a published study protocol [32]. Table 1 describes the eligibility criteria. This population will be referred to as “sample A (primary care, Chiropractic)”.

**Table 1:** Eligibility criteria

Sample	Eligibility criteria	
Sample A (primary care, Chiropractic)	Inclusion	Low back pain 18–65 years of age.
	Exclusion	Verified pregnancy. Serious spinal pathology.
Sample B (primary care, sick listed with risk of chronicity)	Inclusion	Subjects who were considered at high risk of developing chronic disabling low back pain and/or neck pain and long term sick leave using an extensive risk assessment tool.
		- Ongoing sick-listing ≥ 2 months due to low back pain and/or neck pain and no rehabilitation during this sick-listing period.
		Alternatively
		- Recurrent pain and pain at time of examination and sick-listed due to low back pain and/or neck pain ≥ 1 time during the previous year or currently sick-listed due to low back pain and/or neck pain < 2 months.
	Exclusion	Serious spinal pathology.
Sample C (secondary care, multimodal single-center)	Inclusion	LBP and/or NP.
		Cumulative sick-listing for a total of one month to six months during past year (due to low back pain and/or neck pain).
		Fluency in Swedish.
	Exclusion	Previous rehabilitation at the clinic.
		Verified pregnancy.
		Serious spinal pathology.
Sample D (secondary care, multimodal multi-center)	Inclusion	Nonspecific spinal pain.
		Current and continuous sick-listing for at least one month and a maximum of six months before inclusion (due to low back pain and/or neck pain).
		Fluency in Swedish.
	Exclusion	Exposure to physical trauma 6 prior to examination.
		Objective neurological signs indicating need for surgery.
		Co-morbidities (e.g. alcohol abuse, acute psychosis)
		Ongoing rehabilitation.
		Verified pregnancy.
		Serious spinal pathology.

The second sample came from a large intervention study entitled “Work and Health

in the Processing and Engineering Industries” (abbreviated AHA in Swedish) conducted at four companies in Sweden between 2000 and 2003, and is described in detail elsewhere [33, 34]. The purpose of the study was to evaluate an extensive risk assessment tool and an evidence based work place intervention to improve workers’ health. Subjects considered at high risk of developing chronic disabling NP and/or LBP and long term sick leave based on the responses in the screening assessment, were included in this study. This population will be referred to as “sample B (primary care, sick listed with high risk of chronicity)”.

The third and fourth samples came from the HUR project (Health-economic Evaluation and Rehabilitation) that commenced in 1994 with the purpose of evaluating multidisciplinary rehabilitation interventions (at specialized secondary care units) with regards to their effect on sick leave and health related quality of life as well as their cost-effectiveness (herein described as the secondary care populations). The part of the HUR study that focused on NP/LBP was designed as two separate prospective trials, one controlled observational outcome study consisting of subjects with intermittent sickness absence (herein described as “sample C (secondary care, multimodal single-center)”) [35–37] and one randomized controlled trial consisting of subjects with ongoing sickness absence (herein described as “sample D (secondary care, multimodal multi-center)”) [38, 39]. Data were collected as part of the baseline assessment at the initial visit to the clinics. The projects have been described in detail elsewhere [35, 37–39], and Table 1 describes the eligibility criteria.

### Data collection and the MPI-S instrument

The West Haven-Yale Multidimensional Pain Inventory (MPI) has been used to assess patients with a wide variety of chronic pain conditions such as neck pain (NP) and LBP [34, 36, 40], tempero-mandibular disorders [41], headaches [42], fibromyalgia [43] and cancer pain [44] and has been used cross culturally with translations into several languages [45–47]. All four studies used the Swedish version of the MPI (MPI-S) to investigate the psychological and behavioral characteristics of the study populations. The MPI-S is described in previous publications and has been shown to have acceptable reliability and validity [48–50]. Sample D (secondary care, multimodal multi-center) [38, 39] was used in the validation process of the Swedish version of MPI and has been included as a reference sample in this study to ensure reliable estimates.

In short, MPI-S is a 34-item, 8 scales inventory divided into two parts. Part one consists of five scales and is designed to measure important dimensions of psychological factors of pain; pain severity, interference, life control, affective distress and support. Part two consists of 3 scales and is designed to measure behavioral factors of pain; punishing responses, solicitous responses and distracting responses. See Table 2 for a detailed description of the scales in which the text is modified from the original article by Kerns et al. [29].

**Table 2:** Description of the MPI-scales [29]

Dimension	MPI-scales	Description
Psychological	Pain severity (PS)	Perceived pain severity and suffering
	Interference (I)	Perceived pain related life interference, including interference with family and marital functioning, work and work-related activities, and social-recreational activities.
	Life control (LC)	Perceived life control, incorporating the perceived ability to solve problems and feelings of personal mastery and competence.
	Affective distress (AD)	Ratings of depressed mood, irritability and tension.
	Support (S)	Appraisal of support received from spouse, family and significant others - such as worrying, being supportive and attentive.
Behavioral	Punishing responses (PR)	Perceived range and frequency of responses (behaviors) by significant others to displays of pain and suffering by showing frustration, irritation, anger and ignorance.
	Solicitous responses (SR)	Perceived range and frequency of responses (behaviors) by significant others to displays of pain and suffering by helping with medication, food, chores and rest.
	Distracting responses (DR)	Perceived range and frequency of responses (behaviors) by significant others to displays of pain and suffering by such things as involving them in activities, taking their mind off their pain and encouraging them to focus on things other than their pain experience.

*MPI* The Multi-dimensional Pain Inventory

Further, three different subgroups have been empirically derived from the scales through a cluster analytical strategy [50–52] to differentiate patients based on their psychological and behavioral characteristics. The subgroups are named adaptive copers (AC), interpersonally distressed (ID) and dysfunctional (DYS), have been replicated in several studies [31] and are described in additional Table 3. Some authors have added hybrid clusters to adjust for subjects that do not fit perfectly into any of the three suggested subgroups. It was decided not to include these hybrids to allow for better comparison with the reference population [50], and the hybrid subjects were therefore inserted into the closest and most representative cluster.

**Table 3:** Description of MPI-subgroups

MPI-subgroups (abbreviations)	Patient characteristics
Adaptive Copers (AC)	Low pain severity.
	Low interference with everyday life due to pain.
	Low life distress.
	High activity level.
	High perception of life control.
Interpersonally Distressed (ID)	Low levels of social support.
	Low levels of solicitous and distracting responses from significant others.
	High scores on punishing responses compared to the DYS and AC patients.
Dysfunctional (DYS)	High pain severity.
	Marked interference with everyday life due to pain.
	High affective distress.
	Low perception of life control.
	Low activity level.

Both the scales and the subgroups have been used to quantify aspects of the pain experience. The MPI-subgroups are thought to constitute clinically meaningful patient groups. Information about subgroup assignment could be useful for treatment planning, descriptive or evaluative purposes. The patient groups have been investigated with regards to treatment outcome [53–55] and sick leave [34, 36] in LBP patients and have been found to have predictive value and clinical relevance. What represents a clinically important difference in the scales on the MPI instrument is not well established and probably differs between populations, diseases and scales [56]. However, based on normative data, for pain and functioning, a difference of 0.6 on the interference scale represents a clinically relevant difference between populations [56].

### Data analysis

**Fig. 1:**
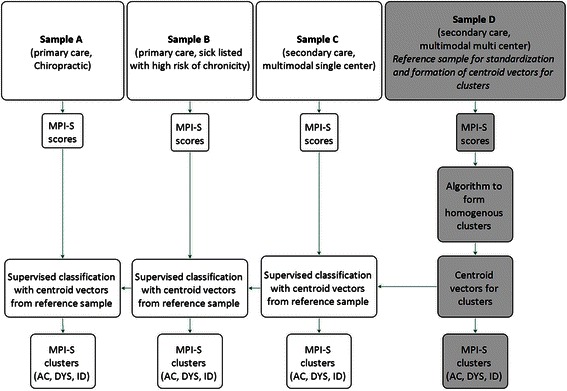
Flowchart describing workflow and formation of clusters. Legend: MPI-S = The Multi-dimensional Pain Inventory - Swedish version, AC = adaptive coper, ID = interpersonally distressed, DYS = dysfunctional

The data were analyzed using SPSS (Statistical Package for the Social Sciences) v20 [57]. Differences in the MPI scales between the samples were analyzed with one-way analysis of variance (ANOVA) starting with an overall test for differences between the samples followed by post-hoc tests where the study population A was contrasted with each one of the study populations B, C and D according to Dunnett’s *t*-test (to avoid significance due to multiple testing). The comparison of the distributions of the MPI-S subgroups in each of the study populations started with a derivation phase where one study population, sample D (secondary care, multimodal multi-center) [38, 39], acted as the reference and the subjects of the other study populations were classified relative to this reference. The method used in this phase was a non-hierarchical cluster procedure (K-Means algorithm). The computations started with a standardization of the MPI-S scales using the mean values and standard deviations of the reference group to form Z-scores and then T-scores. For the reference group a complete cluster analysis was then done to create the centroid vectors (mean values of the MPI-S scales) for the three MPI subgroups AC, ID and DYS. Using these centroid vectors the subjects of the remaining study populations A, B and C, were classified into the MPI-S subgroups but now using only the first initial step of the cluster algorithm, that is, only one iteration in a straight-forward classification, a classify sub-procedure in the K-Means algorithm. Thus the structure of the MPI-S subgrouping from the reference group was applied to the remaining study populations and distributional differences and similarities between all four study populations could be examined. Figure 1 gives a visual description of the processes.

A replication process using a somewhat different statistical approach followed the derivation phase. To this end a discriminant analysis was used with sample D (secondary care, multimodal multi-center) [38, 39], and its MPI-S subgroup structure acting as reference. The subjects of the other study populations were classified in the MPI-S subgroups in accordance with the discriminant function formed from the reference population. In the end the classifications from the cluster approach and the discriminant approach were compared by cross-tabulations and weighted kappa calculated as a measure of agreement.

Finally the distributions of the MPI-S subgroups for the four study populations were compared with a chi-square test for distributional differences.

### Ethical approval

The current study was approved by the local Ethical Committee at the Karolinska Institutet: 2015/1483-32.

The ongoing RCT from which sample A (primary care, Chiropractic) was collected was approved by the local Ethical Committee at the Karolinska Institutet: 2007/1458-31/4, and was registered at Clinical trials.gov; NCT01539863. Written informed consent was obtained from all subjects. The study from which sample B (primary care, sick listed with high risk of chronicity) was collected was approved by the local Ethical Committee at the Karolinska Institutet: (AHA; Dnr 00–012). Written informed consent of each of the employees was obtained.

The studies from which sample C (secondary care, multimodal single-center) and D (secondary care, multimodal multi-center) were collected were approved by the local Ethical Committee at the Karolinska Institutet (Dnr 94:340) and written informed consent was obtained from all study subjects.

## Results

In sample A (primary care, Chiropractic) [32], 480 subjects fulfilled the inclusion criteria and completed the MPI-S questionnaire (81 % of these subject were self-funded, 19 % were partially or fully paid for by third party payers). The corresponding number of subjects who were eligible and included in the other samples were 128 (sample B (primary care, sick listed with high risk of chronicity) [33, 34]), 273 (sample C (secondary care, multimodal single-center) [35–37]) and 235 (sample D (secondary care, multimodal multi-center) [38, 39]). In total, 954 subjects (86 %) had sufficiently complete data to be classified using the cluster analysis. Descriptive data of the study samples are reported in Table 4.

**Table 4:** Descriptive data of the four study samples

Variable		Sample A	Sample B	Sample C	Sample D
n		480	128	273	235
Women	%	53	10	48	55
Age	Mean	45^a^	42	42	43
	S.D.	12.5	9.4	9.5	10.4
Pain duration, months	Mean	N.A.	N.A.	37	32
	S.D.	N.A.	N.A.	60	59
Neck/thoracic pain	%	N.A.	72^b^	32^e^	41^e^
LBP	%	96	94^b^	66^e^	46^e^
Mixed pain sites	%	57	66	2^f^	13^f^
Pain radiating into leg	%	21	N.A.	N.A.	N.A.
Have had LBP episode before	%	75^c^	N.A.	N.A.	N.A.
Have had LBP >30 days previous year	%	66^d^	N.A.	N.A.	N.A.
Population		Primary care, Chiropractic	Primary care, sick listed with risk of chronicity	Secondary care, multimodal single center	Secondary care, multimodal multi center

^a^N = 134, age was recorded at the second baseline in the RCT, therefore data could be recorded only in a minority of the population

^b^Having experienced once or several times during past year

^c^N = 467, ^d^N = 407

^e^NP or LBP as primary pain sites

^f^Mixed as primary pain site, S.D. – Standard Deviation, N.A. – not available, LBP – Low Back Pain

With respect to the MPI-S interference scale, sample A (primary care, Chiropractic) was significantly different to the other samples with scores between B (primary care, sick listed with high risk of chronicity, which had less interference, difference of 0.41) and the secondary samples C (secondary care, multimodal single-center) and D (secondary care, multimodal multi-center) (which had more interference, with a difference of 0.91 and 0.89 respectively). With the previously suggested level for clinically relevant difference of 0.6, this result indicates that the differences from the two samples from secondary care C (secondary care, multimodal single-center) and D (secondary care, multimodal multi-center) were also clinically important.

When comparing sample A (primary care, Chiropractic) to sample B (primary care, sick listed with high risk of chronicity) there were, other than on the interference scale, also significant differences on four other scales. The latter population had lower pain severity, higher life control, lower affective distress and higher degrees of punishing responses. Sample B (primary care, sick listed with high risk of chronicity) also reported a higher frequency of behaviors from significant others displaying anger, frustration and unresponsiveness, when compared to sample A (primary care, Chiropractic).

Both the secondary care samples C (secondary care, multimodal single-center) and D (secondary care, multimodal multi-center) had significantly higher scores on the support and solicitous responses compared to sample A (primary care, Chiropractic). Sample D (secondary care, multimodal multi-center) also scored significantly higher on three other scales (pain severity, punishing responses and distracting responses) when compared to A (primary care, Chiropractic).

The results from the MPI-S scales do not support the hypothesis that sample A (primary care, Chiropractic) is similar or better off than sample B (primary care, sick listed with high risk of chronicity) overall, however some dimensions appear to have similarities. Data are reported in Table 5.

**Table 5:** Comparison of MPI-S scales for the four samples. Statistical significance for overall group differences and for group-wise comparisons with the chiropractic primary care population as reference, using Dunnett’s *t*-test

Scale	Samples											
	A (primary care, Chiropractic) (*n* = 361)		B (primary care, sick listed with risk of chronicity) (*n* = 128)			C (secondary care, multimodal single-center) (*n* = 253)			D (secondary care, multimodal multi-center) (*n* = 212)			Overall group differences
	Mean	SD	Mean	SD	*p*	Mean	SD	*p*	Mean	SD	*p*	*p*
PS	3.40	1.23	2.81	1.33	<0.001	3.58	1.15	0.187	3.69	1.13	0.015	<0.001
I	2.91	1.37	2.50	1.34	0.003	3.82	1.10	<0.001	3.80	0.97	<0.001	<0.001
LC	3.37	1.16	3.84	1.09	<0.001	3.20	1.13	0.201	3.14	1.22	0.063	<0.001
AD	2.68	1.41	2.31	1.39	0.041	2.66	1.59	0.997	2.80	1.45	0.671	0.025
S	4.11	1.62	4.23	1.63	0.814	4.69	1.38	<0.001	4.46	1.58	0.030	<0.001
PR	0.87	1.14	1.27	1.33	0.005	1.08	1.21	0.100	1.14	1.30	0.033	0.005
SR	2.78	1.49	2.89	1.43	0.754	3.17	1.00	0.001	3.08	1.05	0.020	0.001
DR	2.84	1.47	2.98	1.57	0.738	3.14	1.62	0.062	3.18	1.62	0.033	0.038

*PS* Pain Severity, *I* Interference, *LC* Life Control, *AD* Affective Distress, *S* Support, *PR* Punishing Responses, *SR* Solicitous Responses, *DR* Distracting Responses

### Cluster classification

**Fig. 2:**
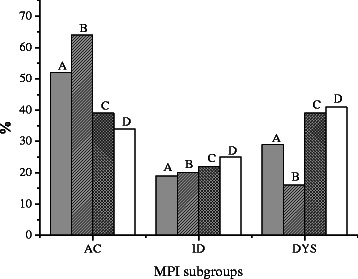
MPI-S subgroups across study samples. Legend: A = study sample A, B = study sample B, C = study sample C, D; study sample D, AC = adaptive coper, ID = interpersonally distressed, DYS = dysfunctional, MP = The Multi-dimensional Pain Inventory

The difference between the four samples was illustrated when comparing the distributions of subgroups within each sample. The proportion of adaptive copers was highest in sample B (primary care, sick listed with high risk of chronicity) followed by sample A (primary care, Chiropractic), C (secondary care, multimodal single-center) and last D (secondary care, multimodal multi-center). The opposite pattern was seen with the dysfunctional subgroup with the order D (secondary care, multimodal multi-center), C (secondary care, multimodal single-center), A (primary care, Chiropractic) and last B (primary care, sick listed with high risk of chronicity). Likewise, a smaller decreasing trend was present for the ID cluster (with the order: D (secondary care, multimodal multi-center), C (secondary care, multimodal single-center), B (primary care, sick listed with high risk of chronicity) and A (primary care, Chiropractic)). Significant differences (sample A (primary care, Chiropractic) as a reference) were observed between samples overall within the AC and DYS cluster, but not within the ID cluster. The difference is most significant between sample A (primary care, Chiropractic) and the two secondary care samples C (secondary care, multimodal single-center) and D (secondary care, multimodal multi-center). This illustrates the difference between the samples with regards to the AC and DYS clusters and does not support the hypothesis that sample A (primary care, Chiropractic) is similar to sample B (primary care, sick listed with high risk of chronicity). Data are reported in Table 6 and Fig. 2.

**Table 6:** The four samples stratified on the three MPI subgroups. A global test of differences in the distribution of MPI subgroups supplemented with posteriori tests of differences from the reference group A

MPI subgroup	Samples			
	A (primary care, Chiropractic) *n* = 361	B (primary care, sick listed with risk of chronicity) *n* = 128	C (secondary care, multimodal single-center) *n* = 253	D (secondary care, multimodal multi-center) *n* = 212
AC	189 (52 %)	82 (64 %)	98 (39 %)	72 (34 %)
ID	67 (19 %)	25 (20 %)	56 (22 %)	52 (25 %)
DYS	105 (29 %)	21 (16 %)	99 (39 %)	88 (41 %)
Study populations differ on MPI group distribution, p < 0.001				
	Reference	A vs B, *p* = 0.048*	A vs C, *p* = 0.009*	A vs D, *p* < 0.001*

*ID* Interpersonally distressed, *DYS* Dysfunctional, *AC* Adaptive Copers

* = *p*-values adjusted for multiple testing

### Validation of cluster classification method

The reproducibility of the cluster solution with a discriminant analysis achieved kappa values above 0.9 for all four populations and was considered excellent [58, 59] according to Cohen’s kappa agreement. The initial analysis using the cluster classify method was thus considered valid. There was, however, a small discrepancy between the two methods, as 33 subjects (3.5 %) were classified differently. Most likely, these subjects should have been described as hybrids, but as the 3 cluster solution was used, they were inserted into the closest neighboring cluster, and the two methods have clearly classified these individuals differently, see Table 7. Complete data for the individual cluster distributions between populations and kappa statistics can be obtained from the authors upon request.

**Table 7:** Cluster proportions

Classification method			Sample				Total
			A	B	C	D	
Cluster analysis (K-means)	ID	% (n)	18.6 (67)	19.5 (25)	22.1 (56)	24.5 (52)	21.0 (200)
	DYS	% (n)	29.1 (105)	16.4 (21)	39.1 (99)	41.5 (88)	32.8 (313)
	AC	% (n)	52.4 (189)	64.1 (82)	38.7 (98)	34.0 (72)	46.2 (441)
	Total	% (n)	100.0 (361)	100.0 (128)	100.0 (253)	100.0 (212)	100.0 (954)
Discriminant analysis	ID	% (n)	17.2 (62)	21.1 (27)	22.1 (56)	24.5 (52)	20.6 (197)
	DYS	% (n)	30.5 (110)	16.4 (21)	38.3 (97)	41.5 (88)	33.1 (316)
	AC	% (n)	52.4 (189)	62.5 (80)	39.5 (100)	34.0 (72)	46.2 (441)
	Total	% (n)	100.0 (361)	100.0 (128)	100.0 (253)	100.0 (212)	100.0 (954)

*AC* adaptive coper, *ID* interpersonally distressed, *DYS* dysfunctional

## Discussion

To our knowledge, this is the first study to compare a chiropractic primary care sample to other samples with regards to psychological and behavioral characteristics using the MPI-S instrument. The main strength of the study is the external standardization of the data and the use of independent centroid vectors for the cluster classification allowing for a good comparison of the MPI-S scales and subgroups between the samples. The data are considered reliable and robust due to the validity of the questionnaire, and the results rely on the large samples and the validation of the cluster classification procedure.

The study shows statistically significant overall differences across samples for the cluster distribution and for all 8 scales for psychological and behavioral characteristics. The cluster classifications placed (in terms of the proportions of the AC and DYS subgroups) sample A (primary care, Chiropractic) between B (primary care, sick listed with high risk of chronicity) and the two secondary care samples C (secondary care, multimodal single-center) and D (secondary care, multimodal multi-center), showing significant differences between the cluster proportions. With regards to the scores on the MPI-S instrument it was hypothesized that sample A (primary care, Chiropractic) would be similar to or better off than sample B (primary care, sick listed with high risk of chronicity), however this was only the case in some of the dimensions of the MPI-S scales and as a whole sample A (primary care, Chiropractic) should be considered worse off than sample B (primary care, sick listed with high risk of chronicity).

Overall the differences on the scales are fairly small between populations and one may question the clinical relevance of these differences. However, these small differences on the scales give rise to fairly large differences in the cluster proportions in the AC and DYS clusters which have been shown to be clinically relevant [34, 36, 53–55]. Therefore although small, the differences on the scales should be considered relevant to address.

Subjects in the three samples B (primary care, sick listed with high risk of chronicity), C (secondary care, multimodal single-center) and D (secondary care, multimodal multi-center) were either at risk of developing or already had chronic NP and/or LPB. It is important to note that these samples are not to be considered representative of the general population.

Our data support previous findings [26, 27] that patients from chiropractic primary care, when compared to the secondary care sample, have higher self-rated health and lower functional limitations, less anxiety and less depression, as suggested by the high proportion of AC subjects in sample A (primary care, Chiropractic) compared to the secondary populations C (secondary care, multimodal single-center) and D (secondary care, multimodal multi-center). However, the chiropractic patients (A) are worse off when compared to the other primary care population (B) with almost one-third of patients classified as DYS. This could also explain the previous findings from Australia [28] that suggested a higher degree of co-morbidity and depression among chiropractic patients. Further, the high proportion of AC subjects in sample A (primary care, Chiropractic) suggests a highly adaptive group of patients, less affected by psychological and behavioral problems compared to the secondary samples. Perhaps this makes them more suitable for a predominantly manual treatment regimen with a lower need for psychological and behavioral interventions. Examining treatment outcomes stratified according to MPI-S subgroups is an important future research area. Such data may result in tailored treatment strategies according to psychological and behavioral needs.

Our findings suggest that the subjects in sample B (primary care, sick listed with high risk of chronicity) may be less affected by their LBP symptoms compared to sample A (primary care, Chiropractic). On the other hand, the same population reported a higher frequency of behaviors from significant others displaying anger, frustration and unresponsiveness, when compared to sample A (primary care, Chiropractic). One reason for this difference may be that these characteristics often need a longer period of pain to manifest and given the more acute nature of sample A (primary care, Chiropractic) these have not had enough time to develop.

Logically, samples of subjects with chronic pain (C (secondary care, multimodal single-center) and D (secondary care, multimodal multi-center)), with high pain severity and interference, would be expected to receive support and concern from significant others, which the results support. However the scores in pain severity for sample A (primary care, Chiropractic) were comparable to those of sample C (secondary care, multimodal single-center). At the same time, sample A (primary care, Chiropractic) experienced less support from their significant others (similar to sample B (primary care, sick listed with high risk of chronicity)) yet showed high levels of affective distress. Although a majority (66 %) of the patients in sample A (primary care, Chiropractic) reported more than 30 days of LBP the previous year, it is important to note that they sought care in an acute or sub-acute state, compared to subjects in the other samples which were included in the “steady chronic” state of their condition. This may explain their high pain severity, high affective distress and lower support scores.

A recent study [60] from Switzerland investigated patients with LBP using the German version of the MPI to assess whether the different subgroups responded differently to an intense 4 week multidisciplinary treatment program. This population [60] was characterized by a relatively young age, a high prevalence and a high level of depression, a high level of unemployment, and a long history of pain. The cluster analysis resulted in 29 % DYS, 35 % ID and 32 % AC subjects. This is another example of a population with distinctly different characteristics [60], similar to sample A (primary care, Chiropractic) with regards to the DYS group but very different with regards to the AC and ID clusters.

A methodological consideration may be the co-morbidity of neck pain which is present in varying degrees in all samples. As the inclusion criteria are different and descriptive data are collected differently it is difficult to compare the prevalence of neck pain in the four samples. It is likely that the samples with combined NP and LBP were worse off due to this co-morbidity (as this has been shown to be associated with worse functional status, a poorer prognosis, a less favorable response to treatment [61]). On the other hand recent research has suggested that neck and low back pain in the general population may be regarded as the same disorder in terms of relative prevalence and consequences, regardless of where the pain happens to manifest [62]. However co-morbidity of combined NP and LBP is still an issue that may introduce bias.

The study has some weaknesses that are mainly due to the fact that this is a secondary analysis of data already gathered. First, different descriptive data were collected in the various studies and this limits the comparability of demographic data such as educational level, type of work and socioeconomic status which are important from a bio-psychosocial perspective.

Second, the studies were conducted during different time periods. Data for studies C (secondary care, multimodal single-center) and D (secondary care, multimodal multi-center) were collected about 20 years ago and the most recent study (A (primary care, Chiropractic)) is ongoing. Data collected with such wide time intervals may be subject to bias with regards to differences in social and cultural attitudes to pain that may alter both pain perception and behavior. The acceptance of the bio-psycho-social model has shifted the focus from a strictly patho-anatomical explanation of pain to include psychological and behavioral aspects. In the patho-anatomical paradigm, rest and avoidance of aggravating factors were thought to be important aspects of recovery. Research has found this perspective detrimental to the process of recovery [63]. In the research of etiology and treatment the psychological and behavioral aspects have since been included, and the new paradigm instead emphasizes reassurance, maintenance of activity and engaging in painful activities; to avoid catastrophizing thoughts and to stop perpetuating illness behavior and depression in order to improve self-efficacy and functional status. Therefore, in these 20 years, the way subjects relate to their pain and how their surrounding environment adapts and reacts to their condition may have changed accordingly.

Third, the selection of subjects in sample B (primary care, sick listed with high risk of chronicity) may have introduced bias as the vast majority of the subjects were male (90 %) and blue collar workers (94 %). Previous research has shown that the MPI-S scales has an acceptable internal consistency and construct validity across gender [49]. However there is some evidence to suggest [50] that women are overrepresented in the DYS and ID clusters which may have underestimated the proportion of DYS and ID subjects in sample B (primary care, sick listed with high risk of chronicity).

Fourth, the “healthy worker effect” may also have biased the results. Recent research from Denmark show that chiropractic patients report less sick leave than other populations in primary care [64]. No data on sick leave for the chiropractic population are available in this study, but the mean pain score was higher than in sample B (primary care, sick listed with high risk of chronicity). Thus, the “healthy worker effect” may potentially be present, but it seems unlikely in terms of pain levels and MPI clusters.

Fifth, employment status in sample A (primary care, Chiropractic) and samples C (secondary care, multimodal single-center) and D is unknown. However, employment status is likely to be similar in sample A (primary care, Chiropractic) and B (primary care, sick listed with high risk of chronicity) for two main reasons. One, previous research has indicated higher level of education among chiropractic patients compared to other primary care populations [64]. Two, 82 % of the chiropractic patients in this study payed the full cost for the treatment. Given the high proportion of self-funded individuals most subjects are likely to be gainfully employed. Thus, the risk for such bias when comparing population A and B is regarded as small.

These findings highlight the challenges in generalizations of study results across populations. Even among patients with similar severity of their condition, very different psychological and behavioral characteristics were found between different study populations.

Further, these findings add to the somewhat contradictory knowledgebase regarding the prevalence and importance of psychological and behavioral factors among chiropractic patients and may be used as a reference when comparing research in these settings.

Future research should compare psychological and behavioral factors (using the MPI-S instrument) in other LBP populations in primary care from chiropractors, physiotherapists and family practitioners. This will further inform researchers and clinicians regarding generalizations of results across different populations as well as allow studies of the predictive value of the MPI instrument across different settings.

## Conclusion

This study has described patients seeking chiropractic primary care for LBP with regards to psychological and behavioral characteristics and compared them to the characteristics of patients from a sample at high risk of developing chronic disabling NP and/or LBP and long term sick leave in primary care, and two secondary care samples with chronic LBP. Contrary to the hypothesis the chiropractic primary care sample was more affected by pain and worse off with regards to psychological and behavioral characteristics compared to the other primary care sample. Based on these findings the 4 samples may be considered statistically and clinically different.

## Abbreviations

LBP: Low back pain
NP: Neck pain
MPI-S: Multidimensional Pain Inventory, Swedish version
MPI: Multidimensional pain inventory
AHA: An intervention study entitled “Work and Health in the Processing and Engineering Industries
HUR: An intervention study entitled “Health-economic Evaluation and Rehabilitation”
AC: Adaptive copers
ID: Interpersonally distressed
DYS: Dysfunctional
SPSS: Statistical package for the social sciences
ANOVA: Analysis of variance
